# The role of viral and bacterial infections in the pathogenesis of IPF: a systematic review and meta-analysis

**DOI:** 10.1186/s12931-021-01650-x

**Published:** 2021-02-12

**Authors:** Shayan Mostafaei, Babak Sayad, Maryam Ebadi Fard Azar, Mohammad Doroudian, Shima Hadifar, Ava Behrouzi, Parisa Riahi, Bashdar Mahmud Hussen, Bahareh Bayat, Javid Sadri Nahand, Mohsen Moghoofei

**Affiliations:** 1 Department of Biostatistics, Faculty of Health, Kermanshah University of Medical Sciences, Kermanshah, Iran; 2 Inflammation Research Center, Tehran University of Medical Sciences, Tehran, Iran; 3 Infectious Diseases Research Center, Kermanshah University of Medical Sciences, Kermanshah, Iran; 4 Student Research Committee, Iran University of Medical Sciences, Tehran, Iran; 5 Department of Cell and Molecular Biology, Faculty of Biological Sciences, Kharazmi University, Tehran, Iran; 6 Department of Mycobacteriology and Pulmonary Research, Pasteur Institute of Iran, Tehran, Iran; 7 Department of Biostatistics, Faculty of Medical Sciences, Tarbiat Modarres University, Tehran, Iran; 8 Department of Pharmacognosy, College of Pharmacy, Hawler Medical University, Erbil, Iraq; 9 Department of Microbiology, Faculty of Medicine, Iran University of Medical Sciences, Tehran, Iran; 10 Department of Virology, Faculty of Medicine, Iran University of Medical Sciences, Tehran, Iran; 11 Department of Microbiology, Faculty of Medicine, Kermanshah University of Medical Sciences, Kermanshah, Iran

**Keywords:** Viral infection, Bacterial infection, Idiopathic pulmonary fibrosis, Epidemiology, Meta-analysis

## Abstract

**Background:**

Idiopathic pulmonary fibrosis (IPF) is a chronic progressive lung disease. Several risk factors such as smoking, air pollution, inhaled toxins, high body mass index and infectious agents are involved in the pathogenesis of IPF. In the present study, this meta-analysis study investigates the prevalence of viral and bacterial infections in the IPF patients and any possible association between these infections with pathogenesis of IPF.

**Methods:**

The authors carried out this systematic literature review from different reliable databases such as PubMed, ISI Web of Science, Scopus and Google Scholar to December 2020.Keywords used were the following “Idiopathic pulmonary fibrosis”, “Infection”, “Bacterial Infection” and “Viral Infection”, alone or combined together with the Boolean operators "OR”, “AND” and “NOT” in the Title/Abstract/Keywords field. Pooled proportion and its 95% CI were used to assess the prevalence of viral and bacterial infections in the IPF patients.

**Results:**

In this systematic review and meta-analyses, 32 studies were selected based on the exclusion/inclusion criteria. Geographical distribution of included studies was: eight studies in American people, 8; in European people, 15 in Asians, and one in Africans. The pooled prevalence for viral and bacterial infections w ere 53.72% (95% CI 38.1–69.1%) and 31.21% (95% CI 19.9–43.7%), respectively. The highest and lowest prevalence of viral infections was HSV (77.7% 95% CI 38.48–99.32%), EBV (72.02%, 95% CI 44.65–90.79%) and Influenza A (7.3%, 95% CI 2.66–42.45%), respectively. Whereas the highest and lowest prevalence in bacterial infections were related to Streptococcus sp. (99.49%, 95% CI 96.44–99.9%) and Raoultella (1.2%, 95% CI 0.2–3.08%), respectively.

**Conclusions:**

The results of this review were confirmed that the presence of viral and bacterial infections are the risk factors in the pathogenesis of IPF. In further analyses, which have never been shown in the previous studies, we revealed the geographic variations in the association strengths and emphasized other methodological parameters (e.g., detection method). Also, our study supports the hypothesis that respiratory infection could play a key role in the pathogenesis of IP.

## Background

Idiopathic pulmonary fibrosis (IPF) is a chronic progressive lung disease of unknown etiology**.** IPF causes progressive scar tissue which gets worse over time resulting in acute dyspnea [1, 2]. Alveolar epithelial injury in IPF leads to fibroproliferation, myofibroblast differentiation and excessive collagen and extracellular matrix deposition, causing impairment of gas exchange, respiratory failure and death [3].

The prevalence of IPF is 14–27.9 and 1.25–23.4 cases per 100,000 population in the USA and Europe, respectively [4]. The attributable risk of IPF-related morbidity and mortality is associated with aging and occurs more among males than female [1, 4]. Several risk factors are involved in the IPF pathogenesis such as; smoking, high body mass index, toxins (inhaled) and infectious disease [3]. More recently, numerous studies have demonstrated the role of viruses and bacteria in the pathogenesis of IPF. It has been shown that patients with IPF have an increased bacterial load in bronchoalveolar lavage (BAL) fluid compared to healthy people or chronic obstructive pulmonary disease (COPD) patients [5–8]. Furthermore, various viruses and bacteria has been studied in the pathogenesis of IPF, including Respiratory syncytial virus (RSV), Parainfluenza virus (PIV), Rhinovirus, Coronavirus, Cytomegalovirus (CMV), Influenza virus, *Strepococcus, Haemophillus* and *Neisseria* [6, 9–11]. It has been reported that inflammation plays a critical role in genesis and progression of IPF in both human and murine models, indicating that viral and bacterial infections can be led to chronic infection and inflammation that maybe are the cause of IPF [12, 13].

Although several studies have been conducted to determine the prevalence of viral and bacterial infection in the IPF patients, the association between IPF pathogenesis and viral/bacterial infection remains the subject of ongoing investigation. This meta-analysis study investigates the prevalence of viral and bacterial infections in the IPF patients and any possible association between these infections with pathogenesis of IPF.

## Methods

### Search strategies

In this meta-analysis, a systematic search was conducted for previous studies relevant (2020) reliable databases, ISI Web of Science, PubMed, and Scopus. Literature searches were carried out by using the following keywords “Idiopathic pulmonary fibrosis”, “Infection”, “Bacterial Infection” and “Viral Infection”, alone or combined together with the Boolean operators "OR”, “AND” and “NOT” in the Title/Abstract/Keywords field. It should be noted that unpublished studies were not included, and duplicate ones were removed. Our literature searches were conducted by three reviewers independently and the search results were compared to prevent having missing data. Also, we screened citations of collected papers to identify additional eligible studies. Titles, abstracts and keywords field of all papers were screened, and unrelated studies were excluded to increase of specificity in the search results. This study was registered in PROSPRO (ID: 170736).

### Inclusion and exclusion criteria

The inclusion criteria for eligible publications were defined as: all research that

- Published: 1990 to 2020.
- Reporting the presence of viral or/and bacterial infections [previous (colonization) and/or new infection] in IPF patients.
- Conducting valid laboratory techniques such as: molecular technique, culture, and serology.
- Selecting the proper sampling method including: NPS, OPS, Sputum, Serum, Blood, BAL and Lung Biopsy.

The exclusion criteria: all research that

- Providing incomplete data or failed presented data clearly.
- Animal models-based research.
- Had other infectious agents.
- Overlapping subjects, time, and place of sample collection.

### Data extraction and quality assessment

Data extraction was conducted by two authors separately and independently based on author’s name, year of publication, total sample size, number of bacterial and viral infections patients, country, types of bacteria, types of viruses, types of samples and detection methods. Individual data from each included study were used in this meta-analysis. Extracted data were compared and rechecked by the first and corresponding authors. The methodological quality of the included studies was evaluated using the STROBE checklist. A maximum quality evaluation score of 32 was considered and articles with scores below 18 were excluded from this study [14].

### Statistical methods

Pooled proportion and its 95% CI were used to assess the prevalence of viral and bacterial infections in the IPF patients. Generalized linear mixed and random intercept logistic regression models were used for pooling prevalence [15]. The heterogeneity of proportions between included studies was tested and quantified by using Cochran's Q test, Tau^2 and I2, respectively [16, 17]. The maximum-likelihood estimator was employed to estimate Tau^2. Logit transformation and Clopper-Pearson were used for pooled proportion and confidence interval in the individual studies. Also, continuity correction of 0.5 in studies with zero cell frequencies [18]. The pooled proportion, as an overall prevalence of viral and bacterial infections in IPF patients was derived by a random effects model because of significantly heterogeneity between the individual studies. However, influence analyses was performed by the Baujat plot which is a diagnostic plot to detect studies contributing to the heterogeneity of a meta-analysis [19]. A funnel plot was conducted to detect publication bias (logit transformed proportions against standard error). Publication bias was tested by Egger’s linear regression and Begg’s tests as it was described (P < 0.05 was considered statistically significance for publication bias) [20]. Finally, the sub-group analyses were used by types of virus and bacteria, year of publication, and country. Meta-regression was applied for assessing the effect of age on the pooled prevalence. All of statistical analyses were performed by using “metafor" and “meta” R packages.

## Results

### Search results and studies characteristics

**Fig. 1:**
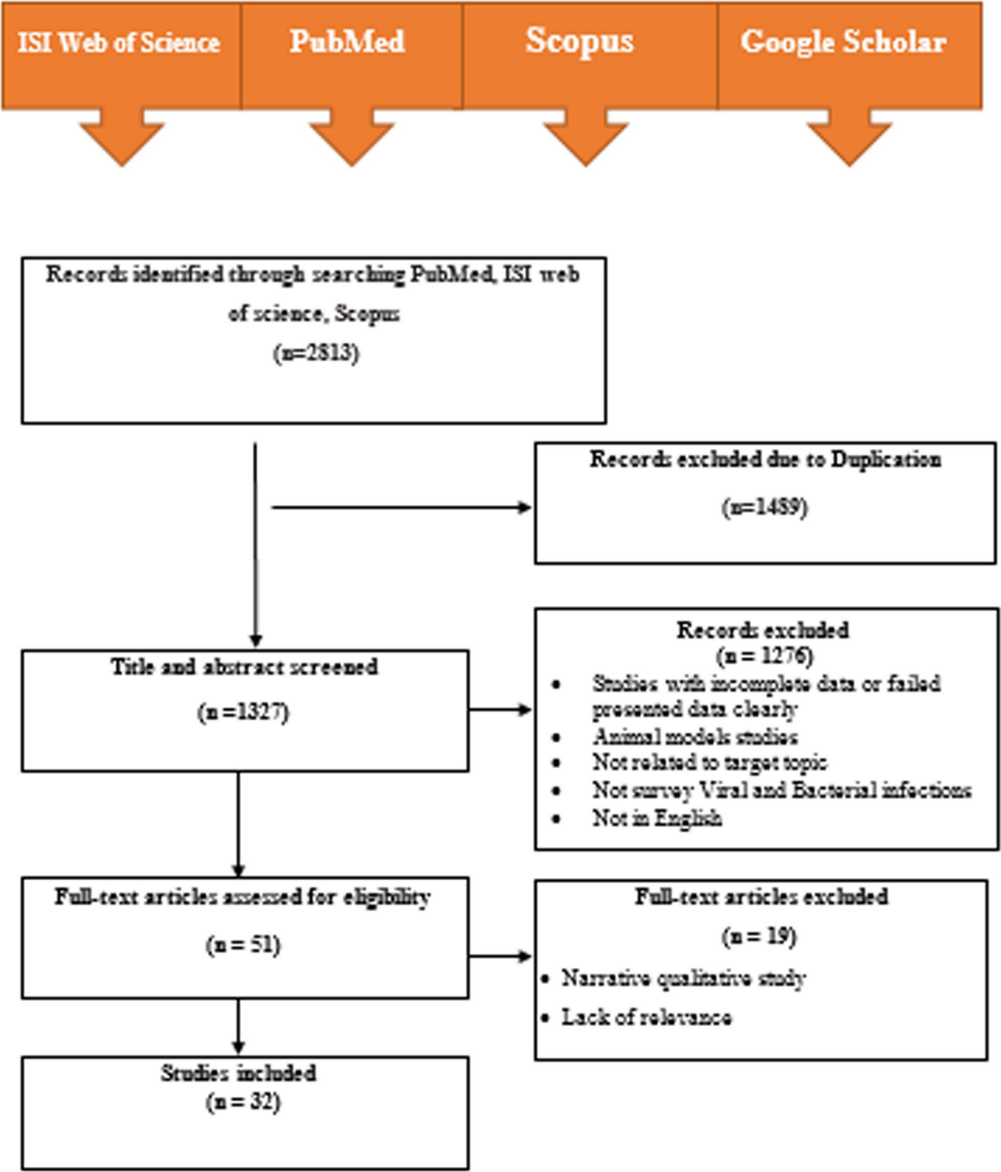
Search flow diagram for 32 studies included in the meta-analysis

The process of research selection shown in Fig. 1 was designed according to Preferred Reporting Items for Systematic Reviews and Meta-Analyses. In the initial search, 2813 articles were identified from ISI Web of Science, PubMed, Scopus and Google scholar databases. Based on the exclusion/inclusion criteria, 32 studies were included in the final meta-analysis. Geographical distribution of included studies was; eight studies in America, eight in Europe, 15 in Asia, and one in Africa. These studies were published from 1992 to 2020 (Table 1).

**Table 1:** Characteristics of 32 included studies in this meta-analysis

First author	Country	Year	Sex	Age	Total	Viral genotypes	N	*Bacterial species*	N	Diagnosis	Method	Sample type	Smoking	Drugs
Ueda [39]	Japan	1992	46 M/20 F	61.5	66	HCV	19	*NA*	*NA*	Clinical, radiologica, physiological, and histological grounds	ELISA, RIBA	Serum	NA	CS
Meliconi [40]	Italy	1996	43 M/18 F	61	60	HCV	8	*NA*	*NA*	Clinical, radiological, physiological, and histological grounds	RT-PCR, ELISA, RIBA	Serum	NA	Immunosuppressive treatment
Kuwano [41]	Japan	1997	8 M/11 F	62	19	Adenovirus	3	*NA*	*NA*	Clinical, radiological, physiological, and histological grounds	Nested PCR,ISH	Lung	Yes	CS
Yonemaru [10]	Japan	1997	30 M/13 F	63	43	CMV,Adenovirus, EBV, HSV, Parainfluenza	42 CMV,Adeno, EBV, HSV /43Parainfluenza 3	*NA*	*NA*	Clinical, radiological, physiological, and histological grounds	EIA,CF, HI	Serum	NA	NA
Stewart [42]	United Kingdom	1999	19 M/8 F	57	27	EBV	13	*NA*	*NA*	UIP pattern at histopathologic examination and HRCT	PCR	Lung	Yes	CyA, Az, Pred, cyclophosphamide
Tsukamoto [43]	Japan	2000	22 M/7 F	57.33	25	EBV	24	*NA*	*NA*	UIP pattern at histopathologic examination	PCR	Lung	Yes	None
Tang [44]	United States	2001	NA	NA	23	EBV,CMV, HHV, HSV/VZV/HHV	7CMV,15EBV,19HHV	NA	NA	UIP pattern at histopathologic examination	PCR	Lung	NA	NA
Lok [45]	United Kingdom	2001	10 M/4 F	59	14	EBV	8	*NA*	*NA*	UIP pattern at histopathologic examination and HRCT	PCR, IHC	Lung	NA	NA
Kelly [46]	United Kingdom	2002	15 M/11 F	52.2	27	EBV	23	NA	*NA*	UIP pattern at histopathologic examination and HRCT	PCR	Serum	NA	CsA/prednisolone/azathioprine
Magro [47]	United States	2003	11 M/8 F	50.9	19	CMV, Parvovirus B19	9 CMV, 9B19	NA	*NA*	UIP pattern at histopathologic examination	IndirectImmunofluorescentRodent Lung Assay	Serum- lung biopsy	NA	NA
Tang [48]	United States	2003	23 M/10 F	55.27	33	CM, HHV, EBV	21EBV, 7CMV, 29HHV	NA	*NA*	UIP pattern at histopathologic examination	PCR	Lung	NA	Prednisone,CytoxanImuran,CytoxaNeoralActimmune
Dworniczak[49]	Poland	2004	9 M/7 F	40.9	16	CMV	12	NA	*NA*	ATS/ERS statement 2000	Q-PCR	BALFBlood-Serum	Yes	Never treated
Miyake [50]	Japan	2005	94 M/10 F	NA	104	HCV	7	NA	*NA*	ATS/ERS statement 2002	NA	Medicalhistory	Yes	NA
Lawson [32]	United States	2008	NA	NA	23	EBV-KSHV- CMV	8EBC, 8CMV, 2KSHV	*NA*	*NA*	ATS/ERS statement2000, ATS/ERS statement 2002	IHC	Lung	NA	NA
Bando [51]	Japan	2008	43 M/14 F	NA	57	TTV	55	*NA*	NA	ATS/ERS statement 2000	PCR	Serum	yes	NA
Pozharskaya[52]	United States	2009	NA	NA	13	EBV	9	*NA*	*NA*	UIP pattern at histopathologic examination	PCR	Lung	NA	NA
Song [21]	South Korea	2011	358 M/103 F	63.4	461	CMV, Influenza, RSV	7CMV, 1Influenza, 1RSV	*S. pneumoniae, MRSA,* *H. influenzae, Legionella, K* *pneumonia,M. tuberculosis*	2S.*pneumoniae,1MRSA,4H.influenzae,1Legionella, 1 K.Pneumoniae, 1 M. tuberculosis*	ATS/ERS statement 2001	Retrospective review	BALF	Yes	Steroid with or without cytotoxic therapy
Wootton [9]	Korea	2011	68 M/15 F	64.06	83	TTV	12	*NA*	*NA*	ATS/ERS statement 2000 Collard et al. 2007	PCR	BALF	Yes	CS with or without immunomodulator therapy
Lasithiotaki [53]	Greece	2011	NA	NA	11 and 13	HHV, HSV	1HSV, 5HHV	*NA*	*NA*	ATS/ERS statement 2000	PCR	Lung, BALF	Yes	NA
Pulkkinen [54]	Finland	2012	11 M/1 F	57.3	12	EBV, HHV,HSV,CMV	11EBV,12HHV,2HSV,2CMV	*NA*	*NA*	ATS/ERS statement 2000, ATS/ERS statement 2002	PCR	Lung	Yes	Prednisolone; cyclosporin A
Calabrese [55]	Canada	2013	39 M/16 F	55.2	55	HHV-6, PV B19, CM, EBV	7HHV, 4CMV, 13EBV	*NA*	*NA*	ATS/ERS statement 2002	PCR, IHC	Lung tissues	Yes	NA
dos Santos [56]	Brazil	2013	7 M/6 F	65	13	MV, CMV	2MV, 1CMV	*NA*	*NA*	ATS/ERS statement 2002	IHC	Surgical lung biopsy	NA	NA
Folcik [57]	USA	2013	14 M/7 F	60.6	21	HVS	21	NA	NA	ATS/ERS/JRS/ALAT statement, Raghu et al 2011	ISH, IHC	Lung	NA	NA
Bando [58]	Japan	2014	5 M/4 F	71.4	9	TTV	9	*Nontuberculus mycobacterium, Streptococcus pneumoniae, Haemophilus influenzae*	3	ATS/ERS/JRS/ALAT statement, Raghu et al. 2012	Real-Time PCR, culture	Serum-sputum	Yes	Steroids/immunosuppressants/ PFD
Ushiki [59]	Japan	2014	11 M/3 F	69.5	14	RSV,CMV	1RSV, 0CMV	*NA*	*NA*	Collard et al. 2007	PCR	BAL	NA	Not treated
Rabea [60]	Egypt	2015	13 M/17 F	52.4	30	HCV	9	*NA*	*NA*	ATS/ERS statement2002	ELISA	Serum	Yes	NA
Keyvani [24]	Iran	2017	22 M/18 F	66.62	40	RSV, Parainfluenza, Rhino, Corona, Influenza	1RSV, 3 parainfluenza,4 rhino,1corona,0 influenza	*NA*	*NA*	CT scan	DNA array assay	Nasopharyngeal, BAL	NA	NA
Saraya [61]	Japan	2018	18 M/9 F	74	27	HHV,HPIV,CMV	3 HHV, 2CMV, 1HPIV	*NA*	*NA*	Collard et al. 2007, Collard et al. 2016	PCR	Nasal swab, sputu, BALF	Yes	Antifibroticagent/CS oral /CSpulseintravenous CY/oral + CsA/CY
Weng [62]	China	2019	NA	NA	149	CMV, Adenovirus, Influenza B, RSV	2CMV, Adenovirus, 4Influenza B, 6RSV	*Mycoplasma, Legionella, Chlamydia*	*11,Mycoplasma, 8Legionella, 1Chlamydia*	ATS/ERS/JRS/ALAT statement, Raghu et al. 2011	IgM ELISA	Serum	Yes	Glucocorticoids, antibiotics, glutathione, immune support therapy
Weng [62]	China	2019	AEIPF48M/0 F StableIPF110M/12 F	AE-IPF = 65 stable IPF = 64	170	NA	NA	*Klebsiella pneumoniae, Acinetobacter baumannii, Mycobacterium* *tuberculosis, Pseudomonas aeruginosa, Serratia marcescense, Raoultella*	*10 K. pneumoniae,4A.baumannii, 8 M* *tuberculosis,3P.aeruginosa, 1S.marcescen, 1Raoultella*	ATS/ERS/JRS/ALAT statement, Raghu et al. 2011	Culture	Sputum	Yes	Glucocorticoids, antibiotics, glutathione, immune support therapy
Le Hingrat [63]	France	2020	17 M/2 F	58	19	HHV,CMV,EBV,TTV	15HHV,2CMV,12EBV,12TTV	NA	*NA*	ATS/ERS/JRS/ALAT statement, Raghu et al. 2011	Real-Time PCR	Lung	Yes	PFD, nintedanib, prednisone
Odashima [22]	Japan	2020	497 M/162 F	70.2	659	NA	NA	NTM, *M. tuberculosis*	35NTM, 23 M.tuberculosis	ATS/ERS/JRS/ALATstatement, Raghu et al. 2011	Retrospective cohort	Retrospective cohort	Yes	Steroid,Immunosuppressant
Jafarian [64]	Iran	2020	16 M/13 F	58	29	EBV, HHV	6EBV,3HHV	NA	NA	ATS/ERS/JRS/ALAT statement, Wells et al. 2013	PCR	Lung	NA	NA

*CS* corticosteroid, *AZ* azathioprine, *PFD* pirfenidone, *CY* cyclophosphamide

### Quality assessment

Based on the results of the STROBE checklist, highest and lowest score were related to Dworniczak et al. (score = 18) and Keyvani et al. (score = 31), respectively. The mean score of STROBE tool for all of included studies was 25.8 (SD = 4.3, range = 18–31). (Table 1).

### Pooled prevalence of viral and bacterial infections in the IPF patients

**Fig. 2:**
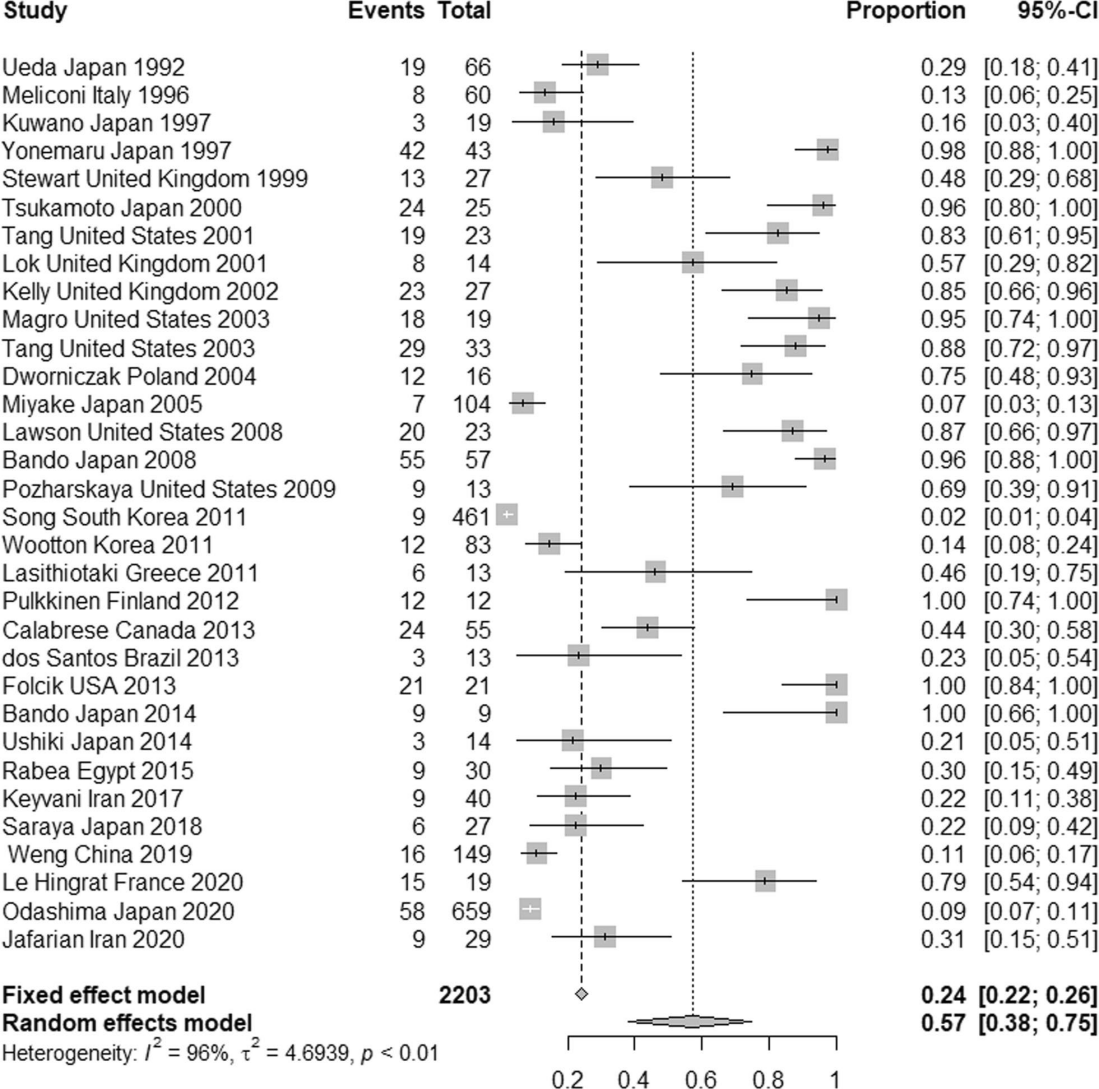
Forest plot of pooled prevalence of viral and bacterial infections in the IPF patients

The total number of the IPF patients included in the study was 2203 individuals aged 26–87 years based on the results of the 32 included studies. The pooled prevalence of viral and bacterial infections in the studied patients was 57.3% (95% CI 37.91–74.75%) according to a random effects meta-analysis. The Wald test showed a significant heterogeneity of prevalence between the studies (Q statistic = 460; Wald test p-value < 0.001; I^2 = 96.5%; τ^2 = 4.69) (Fig. 2). The pooled prevalence for viral infections was 53.72% (95% CI 38.1–69.1%) according to a random effects meta-analysis. While the pooled prevalence for bacterial infections was 31.21% (95% CI 19.9–43.7%) according to a random effects meta-analysis. There was a significant difference of pooled prevalence between viral and bacterial infections (P-value < 0.001).

### Sub-group analysis and meta-regression

**Fig. 3:**
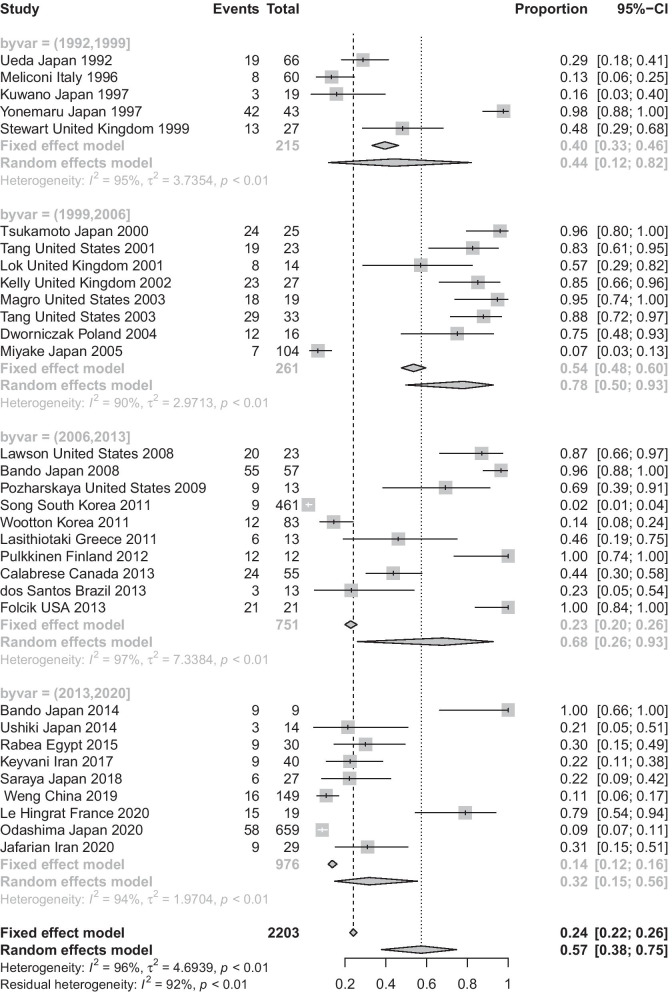
Forest plot of subgroup analysis for the pooled prevalence of viral and bacterial infections in the IPF patients based on the ranges of year of publication

**Fig. 4:**
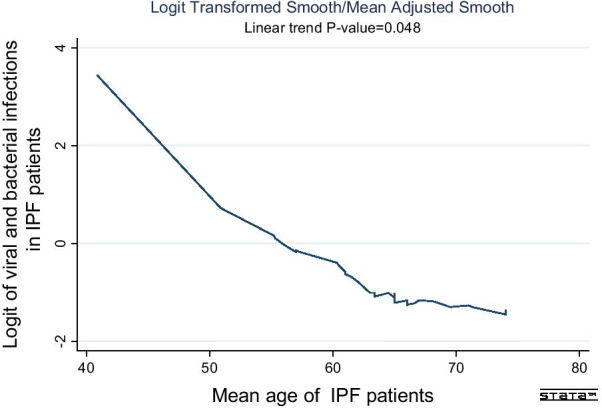
Trend plot of logit (logarithm of odds ratio between the infections and non-infection) of viral and bacterial infections over age of the IPF patients (P-value = 0.048)

The highest and lowest prevalence of viral infections that was reported in patients with IPF as follows; HSV (77.7% 95% CI 38.48–99.32%), EBV (72.02%, 95% CI 44.65–90.79%) and Influenza A (7.3%, 95% CI 2.66–42.45%), respectively (Table 2). Whereas the highest and lowest of this prevalence in bacterial infections were related to Streptococcus sp. (99.49%, 95% CI 96.44–99.9%) and Raoultella (1.2%, 95% CI 0.2–3.08%), respectively. More details about heterogeneity test and publication bias is shown in Table 2. Also, lowest prevalence of viral and bacterial infections was observed in the studies that were published from 2013 to 2020 (32%, 95% CI 15–56%) and the highest of this prevalence related to the studies published between 1999 and 2006 (78%, 95% CI 50–93%). The difference of pooled prevalence between the ranges of year of publication was significant (P-value < 0.001) (Fig. 3). In sub-group analysis based on country, the highest prevalence of viral and bacterial infections was observed in United States (86.9%, 95% CI 65.7–100%) and Japan (69.9%, 95% CI 58.9–78.3%), whereas United Kingdom (5%, 95% CI 2–8.1%) and South Korea (1.5%, 95% CI 0.4–2.6%) showed the lowest prevalence. Based on the meta-regression, with increasing age of the patients, this prevalence was significantly decreased (P-value = 0.048) (Fig. 4). Sub-group analysis based on the type of detection methods showed that PCR technique detected the highest viral infections in IPF patents (76.4%, 95% CI 60.18–90.01%), while using ELISA method showed the lowest prevalence of viral infections (32%, 95% CI 16.1–51.9%). These results for types of detection methods of bacterial infections indicated the highest and lowest prevalence observed in the IPF patients related to sputum culture (60.2%, 95% CI 27.8–92.4%) and ELISA (20.2%, 95% CI 3.3–44.7%) detection methods. In addition, the highest and lowest prevalence of viral and bacterial infections were observed in the IPF patients related to serum (64.8%, 95% CI 38.7–80.9%) and lung tissues/or lung biopsy (33.7%, 95% CI 17.71–41.98%) sample types, respectively.

**Table 2:** Subgroup analysis of the pooled prevalence of viral and bacterial infections in the IPF patients

Virus	No. of studies	Pooled prevalence % (95% C.I) %	Heterogeneity test (I^2^, *P*-value)	Publication bias (Begg's test, P-value; Egger's test, P-value)	Effect model
HCV	3	10.35 (2.29–23.23)	(83.7%; *P-value* = 0.002)	(Begg's Test, 0.30; Egger's test, 0.19)	Random
CMV	12	48.09 (19.53–77.35)	(98.3%; *P-value* < 0.001)	(Begg's Test, 0.73; Egger's test, 0.80)	Random
HSV	4	77.7 (38.48–99.32)	(94.8%; *P-value* < 0.001)	(Begg's Test, 0.73; Egger's test, 0.63)	Random
RSV	3	14.43 (2.51–33.77)	(85.7%; *P-value* < 0.001)	(Begg's Test, 0.30; Egger's test, 0.53)	Random
EBV	9	72.02 (44.65–90.79)	(91.29%; *P-value* < 0.001)	(Begg's Test, 0.66; Egger's test, 0.70)	Random
Adenovirus	2	62.6 (1.0–92.4)	(98%; *P-value* < 0.001)	(Begg's Test, 0.03; Egger's test, 0.17)	Random
Influenza A	2	7.3 (2.66–42.45)	(96.7%; *P-value* < 0.001)	(Begg's Test, 0.53; Egger's test, 0.79)	Random
Parainfluenza	3	48.87 (1.0–99.0)	(98.6%; *P-value* < 0.001)	(Begg's Test, 0.01; Egger's test, 0.13)	Random
B19	2	42.09 (31.28–53.29)	(0%; *P-value* = 0.57)	(Begg's Test, 0.99; Egger's test, 0.58)	Fixed
HHV	8	53.69 (24.52–81.54)	(95.9%; *P-value* < 0.001)	(Begg's Test, 0.79; Egger's test, 0.83)	Random
Rhinovirus	2	15.92 (8.83–25.45)	(62.4%; *P-value* = 0.11)	(Begg's Test, 0.73; Egger's test, 0.57)	Fixed
TTV	3	68.05 (8.19–90.9)	(91.9%; *P-value* < 0.001)	(Begg's Test, 0.68; Egger's test, 0.98)	Random

Bacterial	No. of studies	Pooled prevalence % (95% C.I) %	Heterogeneity test ()	Publication bias (Begg's Test, P-value; Egger's test, P-value)	Effect model
*Mycobacterium tuberculosis*	4	3.16 (0.79–7.01)	(90.27%; *P-value* < 0.001)	(Begg's Test, 0.63; Egger's test, 0.85)	Random
*Haemophilus influenza*	6	8.50 (2.13–18.53)	(86%; *P-value* < 0.001)	(Begg's Test, 0.23; Egger's test, 0.59)	Random
*Streptococcus pneumonia*	5	6.64 (0.6–18.43)	(89.6%; *P-value* < 0.001)	(Begg's Test, 0.13; Egger's test, 0.66)	Random
*Moraxella catarrhalis*	3	5.57 (2.34–10.94)	(0%; *P-value* = 0.97)	(Begg's Test, 0.99; Egger's test, 0.64)	Fixed
*Pseudomonas aeruginosa*	3	2.88 (1.16–5.84)	(0%; *P-value* = 0.41)	(Begg's Test, 0.99; Egger's test, 0.45)	Fixed
*Klebsiella pneumonia*	3	2.94 (0.05–9.92)	(90.6%; *P-value* < 0.001)	(Begg's Test, 0.29; Egger's test, 0.71)	Random
*Streptococcus pneumonia*	5	6.64 (0.6–18.43)	(89.6%; *P-value* < 0.001)	(Begg's Test, 0.43; Egger's test, 0.56)	Random
*Staphylococcus aureus*	3	2.32 (0.02–8.16)	(74.3%; *P-value* = 0.02)	(Begg's Test, 0.08; Egger's test, 0.29)	Random
*Escherichia coli*	2	10.69 (0.05–35.96)	(90.8%; *P-value* = 0.001)	(Begg's Test, 0.18; Egger's test, 0.43)	Random
*Streptococcus* sp.	3	99.49 (96.44–99.9)	(0%; *P-value* = 0.90)	(Begg's Test, 0.99; Egger's test, 0.86)	Fixed
*Serratia marcescens*	2	1.21 (0.22–3.71)	(0%; *P-value* = 0.31)	(Begg's Test, 0.41; Egger's test, 0.83)	Fixed
*Raoultella*	2	1.20 (0.2–3.08)	(0%; *P-value* = 0.31)	(Begg's Test, 0.41; Egger's test, 0.83)	Fixed

### Publication bias and sensitivity analysis

**Fig. 5:**
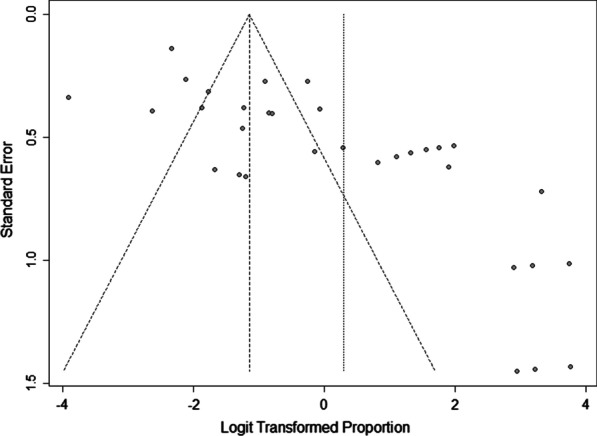
Funnel plot to detect publication bias (Begg’s p-value = 0.041, Egger’s p-value = 0.046)

**Fig. 6:**
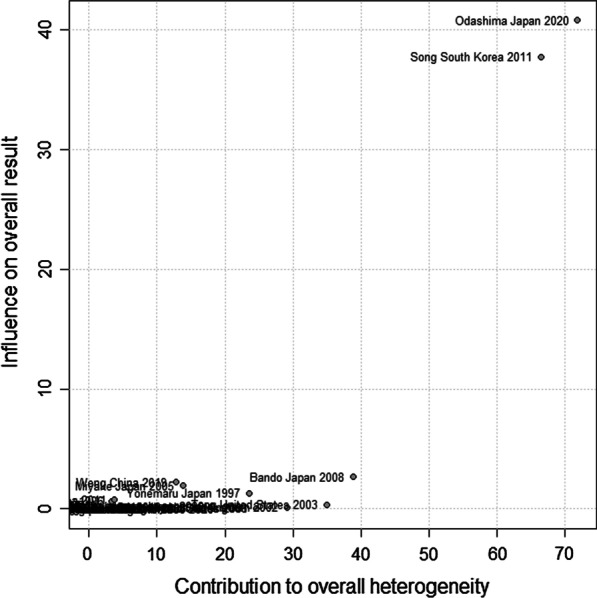
Baujat plot to detect the studies overly contributing to the heterogeneity of this meta-analysis

Publication bias was statistically significant in this meta-analysis (Begg’s p-value = 0.041, Egger’s p-value = 0.046) (Fig. 5). In most of cases in Table 2, the publication bias was not significant. Furthermore, the robustness of the pooled prevalence was checked by Baujat plot as a plot to identify the studies, which overly contributing to the heterogeneity of the meta-analysis. However, the studies of Song et al. in 2011 [21] and Odashima et al. in 2020 [22] have most significant influence on the overall results (P-value < 0.001) (Fig. 6).

## Discussion

IPF is a fatal progressive lung disease that there is no effective cure for this except lung transplant for end-stage IPF patients [23]. Moreover, the FDA approved drugs are associated with a number of side-effects, which compromises their tolerability in IPF patients. Although, the cause of IPF is unknown, recent studies have suggested a strong impact of viral and bacterial infections in both the initiation and progression of IPF may be through aberrant innate immunity [1, 5, 6, 9, 12, 13]. These infectious agents can play a crucial role in increased inflammation and thus in IPF pathogenesis. Viral infection, both lytic and latent, can lead to major fibrosis through increased expression of viral gene products in structural and immune cells in the lung [6, 12, 24]. More recently, a role for bacterial infection has been described in the development of a rapidly progressive clinical phenotype in IPF [5, 25, 26]. Therefore, optimum antiviral and antibacterial immunity in the lung is vital in the maintenance of lung homeostasis and health [5].

Thus far, no systematic review and meta-analysis study has been investigated in the prevalence and the role of viral and bacterial infections in IPF except an investigation by Sheng et al. They suggested viral infection as a risk factor (OR 3.48; 95% CI, 1.61–7.52), however not statistically significant relationship was detected between viral infection and exacerbation of IPF (OR 0.99; 95% CI 0.47–2.12). They also demonstrated that some viruses such as CMV, Epstein-Barr virus (EBV) and human herpesvirus 7, and 8 (HHV-7, HHV-8) were associated with IPF as the risk factors, while HHV-6 was not associated. Therefore, their results indicated that viral infections may involve in IPF pathogenesis [27]. In our study, according to a random effects meta-analysis, the pooled prevalence for viral infections was 53.72% (95% CI 38.1–69.1%) and the highest and lowest prevalence of viral infections was related to HSV (77.7% 95% CI 38.48–99.32), EBV (72.02%, 95% CI 44.65–90.79%) and Influenza A (7.3%, 95% CI 2.66–42.45), respectively (Table 2). Numerous investigations have been conducted on the role of viruses in pathogenesis of IPF, suggesting the role of viral infection in exacerbations of IPF [28]. Wootton et al. studied the detection of viral infection in IPF patients by pan-viral microarray analysis. They detected parainfluenza 1.6% (1/60), rhinovirus 3.3% (2/60) and coronavirus 1.6% (1/60) [9]. In another study, Keyvani et al. assessed forty IPF patients for viral detection and they detected RSV, parainfluenza, rhino and corona viruses in 2.5% (1/40), 7.5% (3/40), 10% (4/40), 2.5% (1/40) and 0% (0/40) of patients, respectively. Their results indicated a significant positive association between age and two viruses (rhinovirus and parainfluenza) [24]. In the current study, based on the type of detection methods of viral infections we indicated that the highest and lowest prevalence of viral infections in previously published studies on IPF were observed by PCR (83.1%, 95% CI 63.75–94.11%) and ELISA (32%, 95% CI 16.1–51.9%), respectively. Thus, it can be proposed that the type of detection method is important in the report of the prevalence of viral infections. Geographical variations might explain the inconsistent results that is present in the studies. In the present investigation, we indicated that the highest prevalence of viral and bacterial infections was in Japan (73.1%, 95% CI 69.3–76.8%) and United States (86.9%, 95% CI 65.7–100%), and the lowest prevalence was in United Kingdom (5%, 95% CI 2–8.1%) and South Korea (1.5%, 95% CI 0.4–2.6%).

The viral infection (especially respiratory viruses) may be involved in increasing of inflammation (chronic), resulting in the pathogenesis of IPF [9, 24, 29]. Due to the function of lung, it is exposed to airborne viruses and there is mounting evidence, which are provided by clinical and preclinical studies, to support a mechanistic role for pathogens of IPF [28]. Some viruses cause latent infections within the alveolar epithelium and under suitable conditions, they are reactivated. This issue can be proposed reactivation of the virus acts as a second hit to the epithelium following exposure to a first injurious insult [30]. Some animal studies have shown that viral infection lead to enhance lung fibrosis hence cofactor in the IPF development [28]. Viral infection induces stress in endoplasmic reticulum (ER) and apoptosis in epithelial cells which can implicate in the development of both IPF and the pulmonary fibrosis [31, 32]. In an investigation, have been shown that acute and chronic viral infections are different in some aspect of Immunopathogenesis [5, 9, 12]. Most of the analyzed studies (including viral infections) in our study were about persistent viruses that were probably acquired before the IPF development.

Although, numerous attempts have conducted on the role of viruses in the IPF pathogenesis, there are a few studies investigating the role of bacteria in this disease. In a recent study, it was demonstrated that 7.5% of IPF patients (BAL sample) were found to be positive in bacterial culture, while none of the controls had bacterial infection. The most common bacterial genus were *Streptococcus* (30%) followed by *Veillonella* (10.6%) and *Prevotella* (10.9%) [6]. In another study, Richter et al. reported 36.3% of stable IPF patients was positive BAL cultures and bacterial genus were *Pseudomonas*, *Haemophilus* and *Streptococcus* [33]. In our study, the pooled prevalence for bacterial infections was 31.21% (95% CI 19.9–43.7%) according to a random effects meta-analysis. In addition, the highest and lowest prevalence in bacterial infections were related to *Streptococcus* sp. (99.49%, 95% CI 96.44–99.9%) and *Raoultella* (1.2%, 95% CI 0.2–3.08%), respectively. High case fatality rate related to bacterial respiratory tract infection in IPF, indicating that bacteria are involved in driving IPF disease progression. Recently, studies using culture-independent techniques have demonstrated that increased bacterial DNA burden in IPF patients is associated with an enhanced risk of earlier mortality in IPF [6, 34, 35]. These results for types of detection methods of bacterial infections showed the highest and lowest prevalence were observed in the IPF patients related to sputum culture (60.2% 95% CI 27.8–92.4%) and ELISA (20.2% 95% CI 3.3–44.7%) detection methods. Also, as mentioned earlier our results indicated the prevalence of viral and bacterial infections was highest in Japan (73.1%, 95% CI 69.3–76.8%) and United States (86.9%, 95% CI 65.7–100%) and this prevalence was lowest in United Kingdom (5%, 95% CI 2–8.1%) and South Korea (1.5%, 95% CI 0.4–2.6%) countries. Infectious agents induce immune responses that can lead to chronic conditions and inflammatory infiltrates, both of which have shown too involved in IPF pathogenesis (23). Moreover, preclinical and clinical studies demonstrated that inflammation is probably involved in initiation and progression of IPF (9, 10).

Cytokine patterns in patients with IPF may shed light on the predominant cell types pivotal to various stages of the disease. Overexpression of Th2 Cytokines including IL-4, IL-5, and IL-13 in cellular cultures from patients with IPF has been previously reported [13]. A series of cytokines (MIP-1α/CCL3), MCP-1/CCL2, and IL-8) connected to neutrophil, monocytes, and lymphocyte chemotaxis and activation are increased significantly in tissue or fluid from the lungs of IPF patients [13, 24]. IL-1α and IL-1β are widely expressed cytokines by alveolar macrophages of IPF patients. This expression lead to induce a pro-fibrotic phenotype through the synthesis of platelet-derived growth factor and procollagen types I and III [36]. Tumor necrosis factor alpha (TNF-α), which is produced by epithelial cells, endothelial cells, lymphocytes and macrophages, upregulates various pathways and factors those involved in inflammation such as the IL-1, IL-6, growth factor beta (TGF-β), C-X-C motif chemokine ligand 8, stimulation of cell–cell adhesion and transendothelial migration [37]. The overexpression of TGF-β results in modulation of extracellular matrix (ECM) productions. This modulation is due to the effects of different factors including fibronectin, proteoglycans, collagens I, III, IV, V and the inhibition of modifying ECM enzymes such as plasminogen and metalloproteinase [38].

All researches that reported the viral and bacterial infection in IPF patients were included in this meta-analysis. The sample size in some researches was small. Furthermore, the viral and bacterial infection rates were variable due to Variety of geographical locations, viral/bacterial detection techniques and the sites of biological samples or type of samples. Different methods led to alterations in sensitivity and specificity. Given these challenges, larger-scale samples are needed in the future to draw conclusions about causal relationship between IPF and viral-bacterial infections.

Our study had several limitations; first, the small sample size, relative wide confidence intervals and study were conducted in a single center. Second, the pathogen types were specific to the study area. Thus, our results are probably not applicable to other patient populations. The association between viral infection and acute exacerbation of IPF requires further investigation.

## Conclusion

The current study provides the overall viral and bacterial infection prevalence in IPF patients and information about circulating types of viruses and bacterial worldwide. The presence of viral and bacterial infections is a risk factor in the pathogenesis of IPF. We revealed the geographic variations in the association strengths and emphasized other methodological parameters (e.g., detection method) in further analyses that have never been shown in the previous studies. Also, our study supports the hypothesis that respiratory infection could play a key role in the pathogenesis of IPF.

## Data Availability

All data generated or analysed during this study are included in this published article and its supplementary information files.

## Abbreviations

IPF: Idiopathic pulmonary fibrosis
BAL: Bronchoalveolar lavage
COPD: Chronic obstructive pulmonary disease
RSV: Respiratory syncytial virus
PIV: Parainfluenza virus
CMV: Cytomegalovirus
EBV: Epstein-Barr virus
ER: Endoplasmic reticulum
TNF-a: Tumor necrosis factor alpha
TGF-β: Transforming growth factor beta
